# Supplementary material for: Amphiphilic Comb Polymers as New Additives in Bicontinuous Microemulsions
Source: Nanomaterials (Basel). 2020 Dec 2;10(12):2410. doi: 10.3390/nano10122410 (PMC7761537; doi:10.3390/nano10122410)
Supplement: Supplementary file 1 [file nanomaterials-10-02410-s001.pdf]

Supporting Information: Amphiphilic comb polymers as new additives in bicontinuous microemulsions

Debasish Saha [1], Karthik R. Peddireddy [2], Jürgen Allgaier [3], Wei Zhang [3], Simona Maccarrone [4], Henrich Frielinghaus [4,*], and Dieter Richter [3]
[1]Solid State Physics Division, Bhabha Atomic Research Centre, Mumbai-400085, India
[2]Department of Physics and Biophysics, University of San Diego San Diego, CA, 92110, USA
[3]Jülich Centre for Neutron Science (JCNS-1) and Institute of Biological Information Processing (IBI-8) ForschungszentrumJülich GmbH, 52425 Jülich, Germany
[4]Jülich Centre for Neutron Science (JCNS), ForschungszentrumJülich GmbH, Outstation at FRM II, Lichtenbergstr. 1, 85747, Garching, Germany

Comb polymer synthesis
The comb synthesis was performed as described in Scheme S1. The procedure prior to the functionalization of the PBO backbone with OH-groups is described in detail in Reference 1, however in this work different ratios of the monomers 1,2-butylene oxide (BO) and 1,2-epoxy-7-octene (EOc) were used. Furthermore, the backbone polymerization reactions were terminated by adding a 5 to 10 fold molar excess of iodomethane compared to the initiator to create -O-CH_3 chain ends. Iodomethane was first degassed and stirred for at least 24h over CaH_2. In some cases, only a fraction of the backbone vinyl groups were oxidized to reduce the number of sidechain in the final combs.

The polymerization of the PEO sidechains was carried out as follows: the backbone materials functionalized with OH-groups were dried for several days under high vacuum conditions in flasks equipped with Teflon stopcocks to exclude contact with air. After redissolution in dry toluene, Potassium metal was added such that about 25 to 33% of the OH-groups were metalated. The mixtures were heated up to 80 °C for up to 2 days until all of the metal had disappeared. Dry ethylene oxide was added to polymerize the sidechains at 100 °C overnight. The ethoxylation reactions were carried out in a steel reactor for safety reasons. The reactions were terminated with acetic acid. After removal of toluene and washing with water, the products were dissolved in chloroform. Finally, the pre-dried polymers were freeze-dried from benzene solutions. Figure S2 shows the SEC traces of the raw Comb 8 product and the corresponding backbone. Further SEC traces are added as Figure S15a-g. All ^{1}H-NMR measurements can be found in Fig. S16a-h.

Polymer characterization
The backbone molecular weights were determined by static light scattering in n-heptane using a DAWN HELEOS light scattering instrument (Wyatt). A Zimm analysis of the data at five different concentrations was then performed. The backbone and comb polymers were further characterized by SEC and ^{1}H-NMR. SEC experiments were carried out using a Polymer Laboratories PL 220 SEC instrument together with five Styragel columns having a porosity range from 10^5 to 500 Å at 30 °C. The solvent was a mixture of THF and N,N-dimethylacetamide (DMA) (90:10 by volume) at a flow rate of 1 mL/min. The remaining experimental details are given in Reference 2. The mass concentration of vinyl groups in the copolymerization products of BO and EOc was determined by ^{1}H-NMR in $CDCl_3$ from the intensities of the vinyl signals at 4.9 ppm and 5.8 ppm and the signal intensity of the internal standard 1,1,1,2,2-pentabromoethane at 6.3 ppm. The mass concentration of OH-groups in the backbone products after the oxidation step was calculated from the difference in vinyl group concentrations before and after the reaction. For the calculations of the number of OH-groups (i.e the number of sidechains per polymer chain), the *tert*-butoxy starter group signal at 0.9 ppm was taken as the reference.

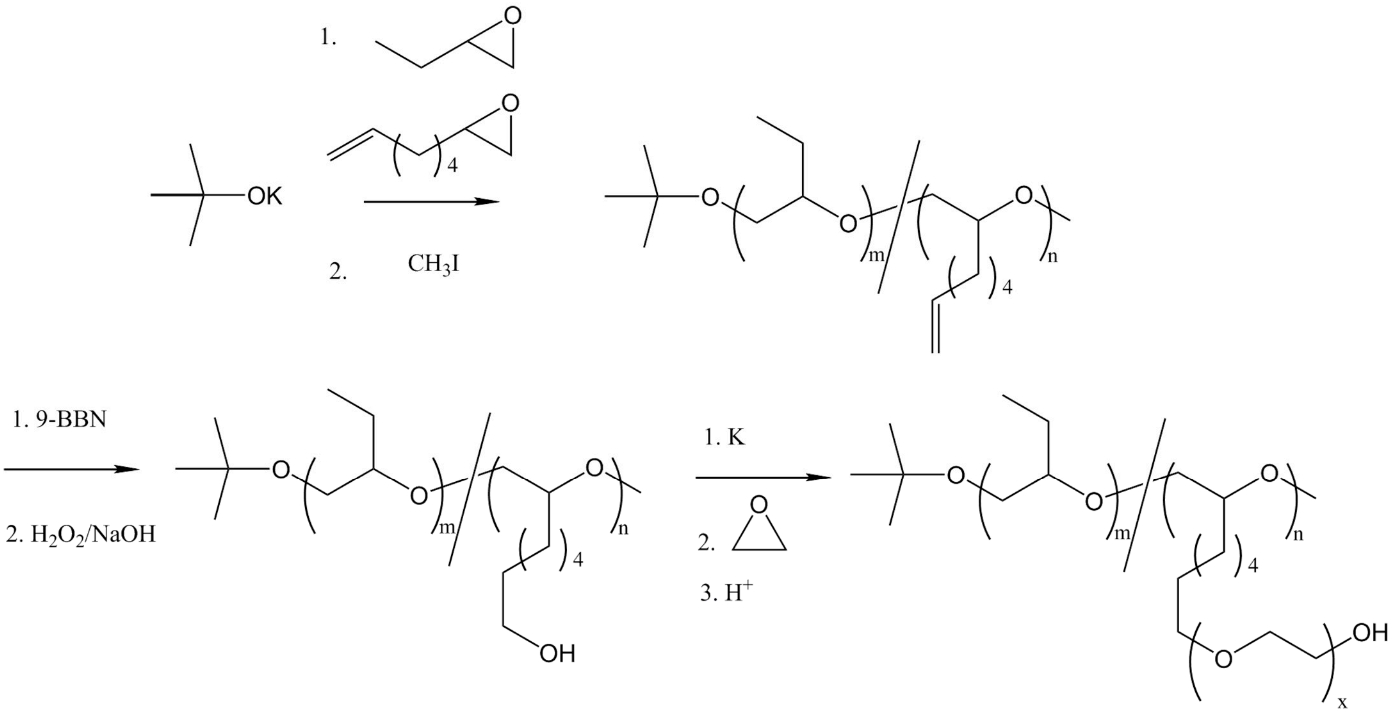

Fig. S1: Scheme of the synthesis of the PBO-PEO comb polymers.

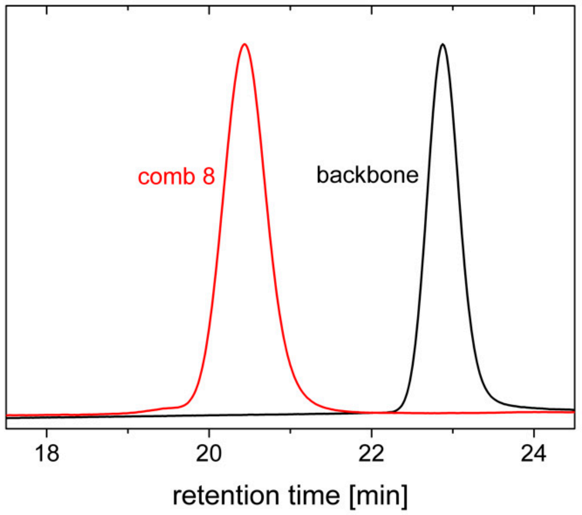

Fig. S2: SEC traces of the non-functionalized backbone and the final comb product for Comb 8. For the comb, the raw product before purification was used. Traces of the other polymers Comb 1-7 are shown in Fig. S15a-g.

In the following figures S3-S10, we present the analysis of the comb polymers 1, 2, 3, 5, 6, 7, 8, and PEG(AOT) in their respective microemulsions. The figures are all labeled as follows: (a), the fish-tail phase diagram (corresponding to Fig. 4 of the main text); (b), the scaling of the efficiency (c.f. Fig. 5); (c), the shift of the phase inversion temperature for the mean curvature (c.f. Fig. 6); (d) and (e), representations of SANS curves with constant surfactant amount and constant polymer amount respectively (c.f. Fig. 7a,b) and (f), the scaling of the bending rigidity obtained from SANS (c.f. Fig. 8b). The scheme (a) to (f) is kept for all different comb polymers (and PEG) to make comparisons easy, even if data for some of the individual plots have not been measured.

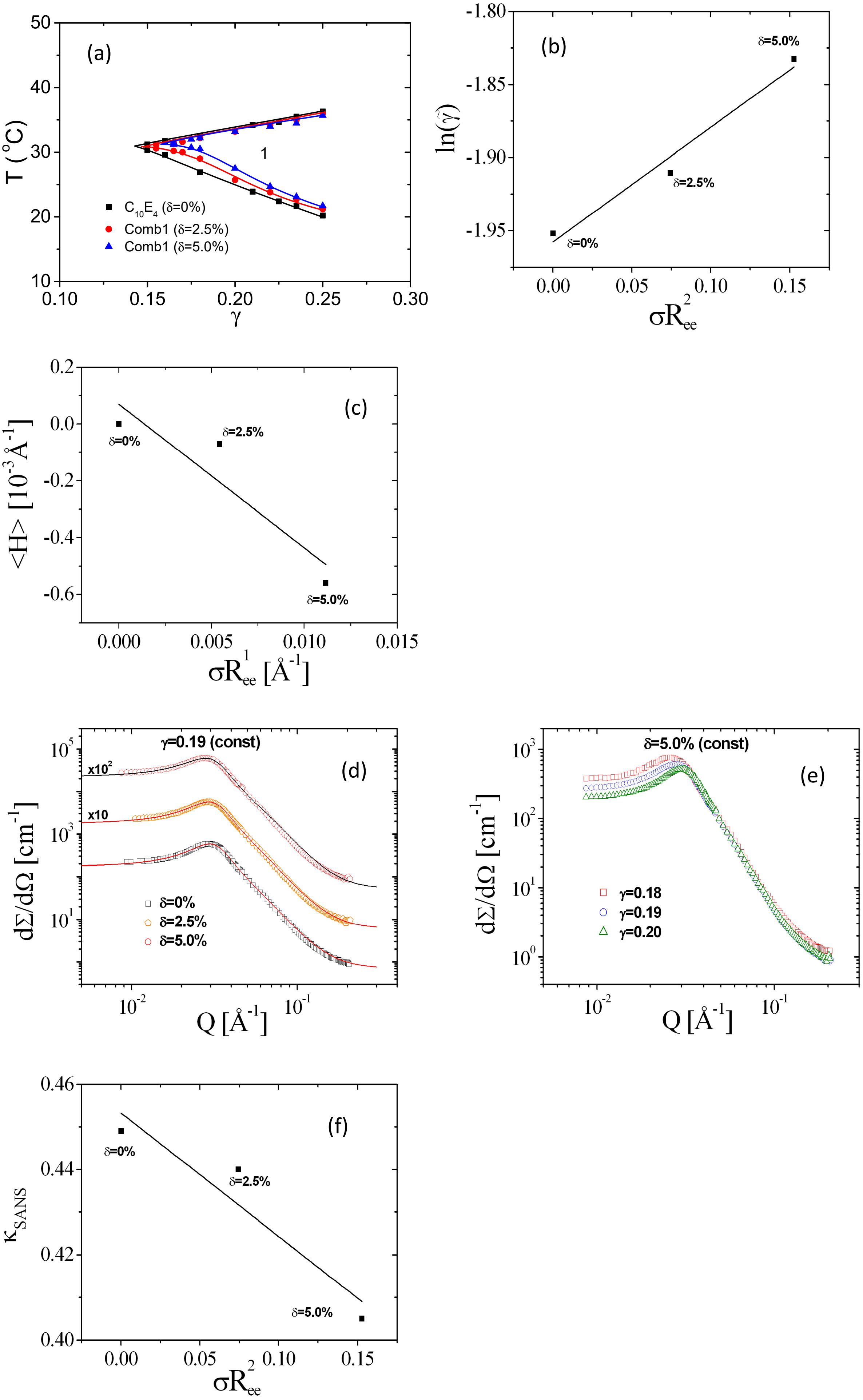

Fig. S3 a-f: Microemulsion characterization with Comb 1.

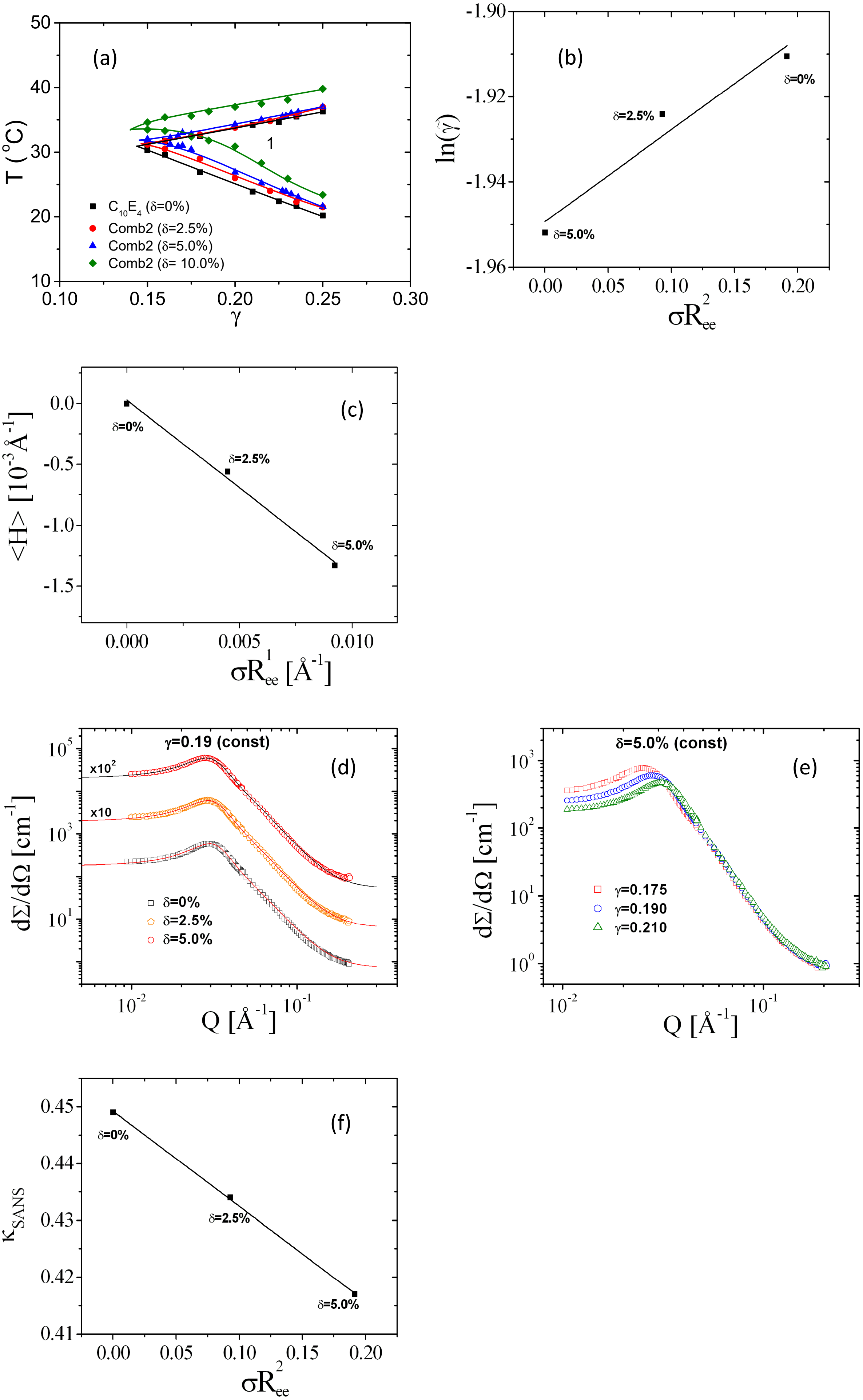

Fig. S4 a-f: Microemulsion characterization with Comb 2 .

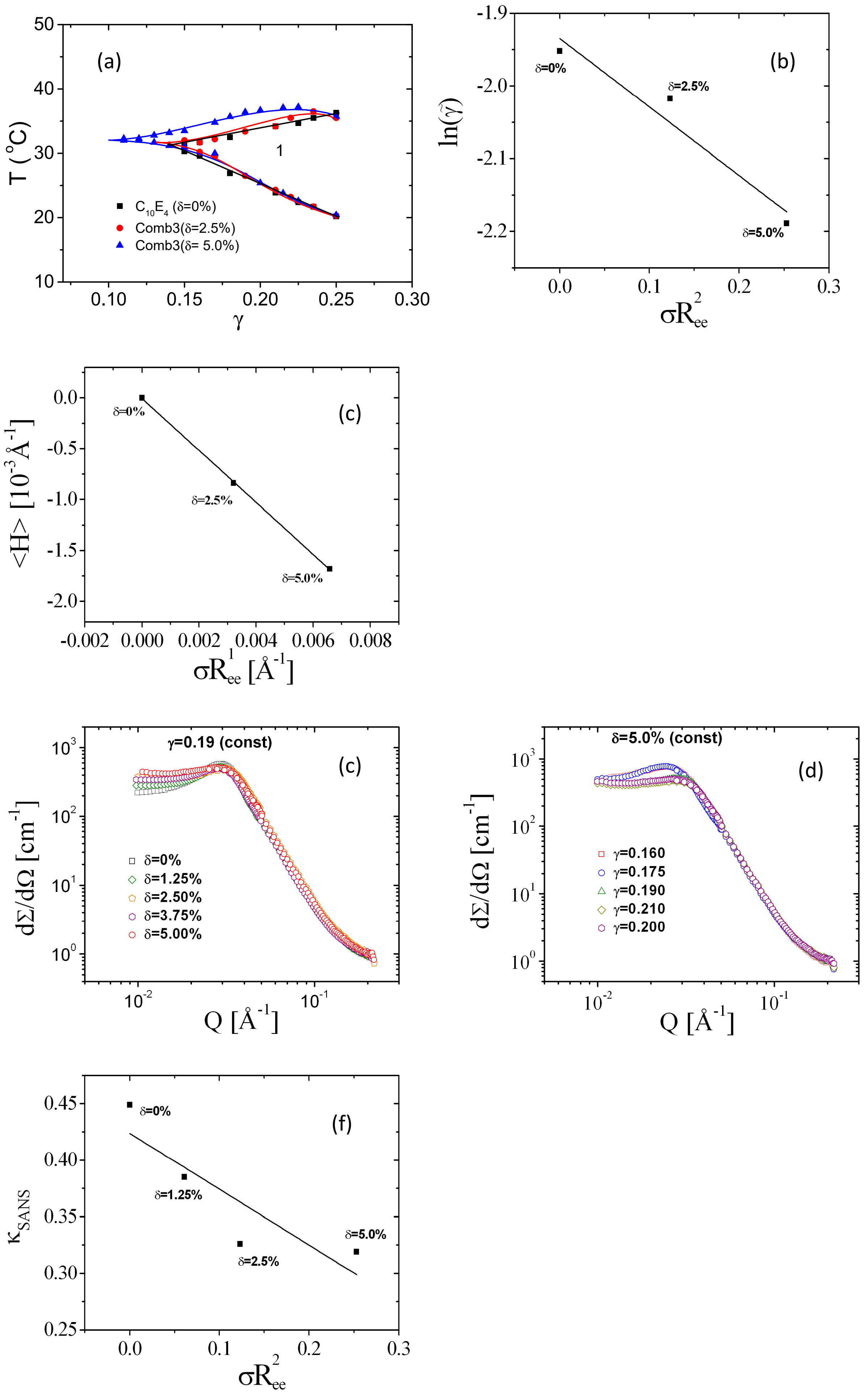

Fig. S5 a-f: Microemulsion characterization with Comb 3.

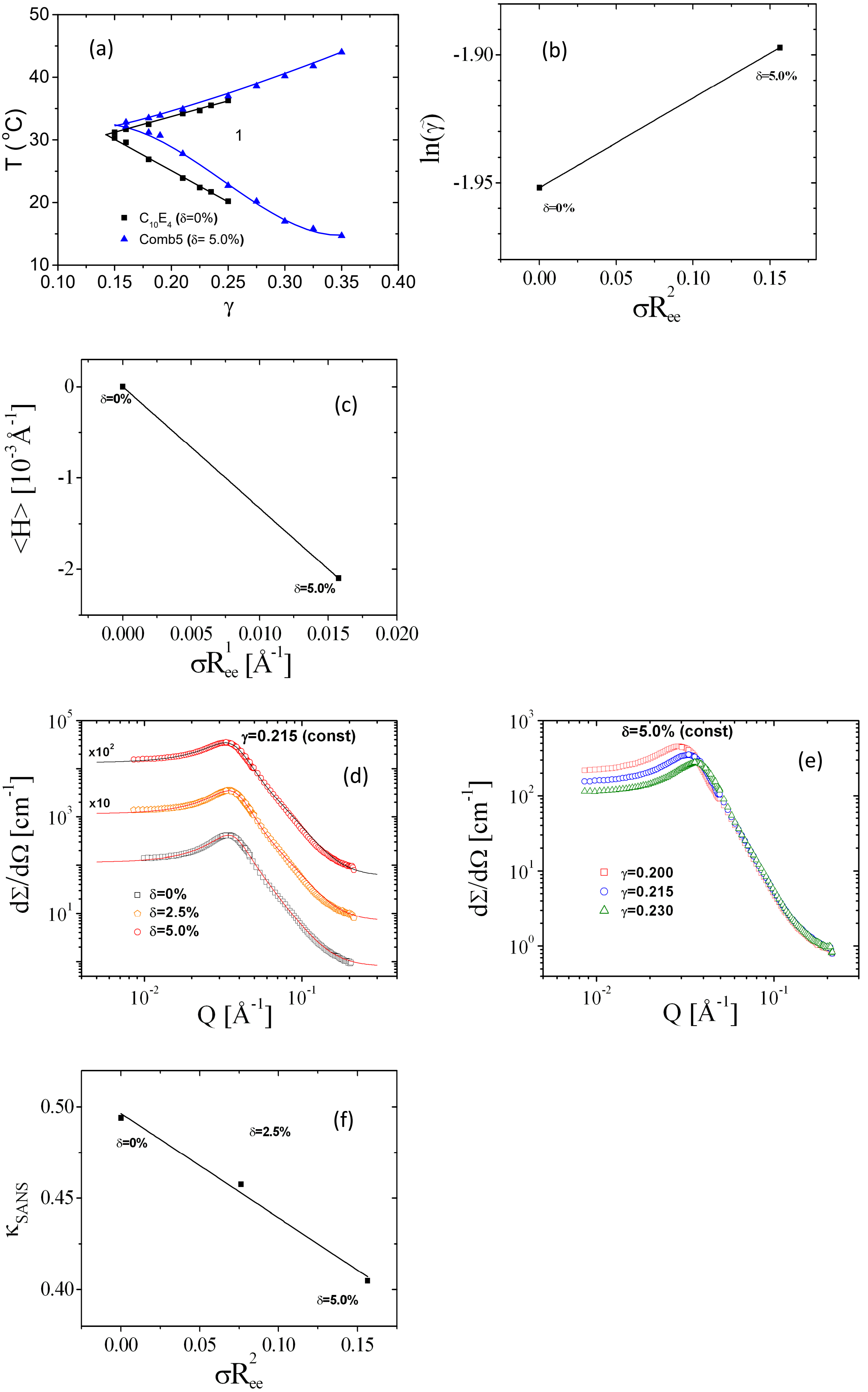

Fig. S6 a-f: Microemulsion characterization with Comb 5.

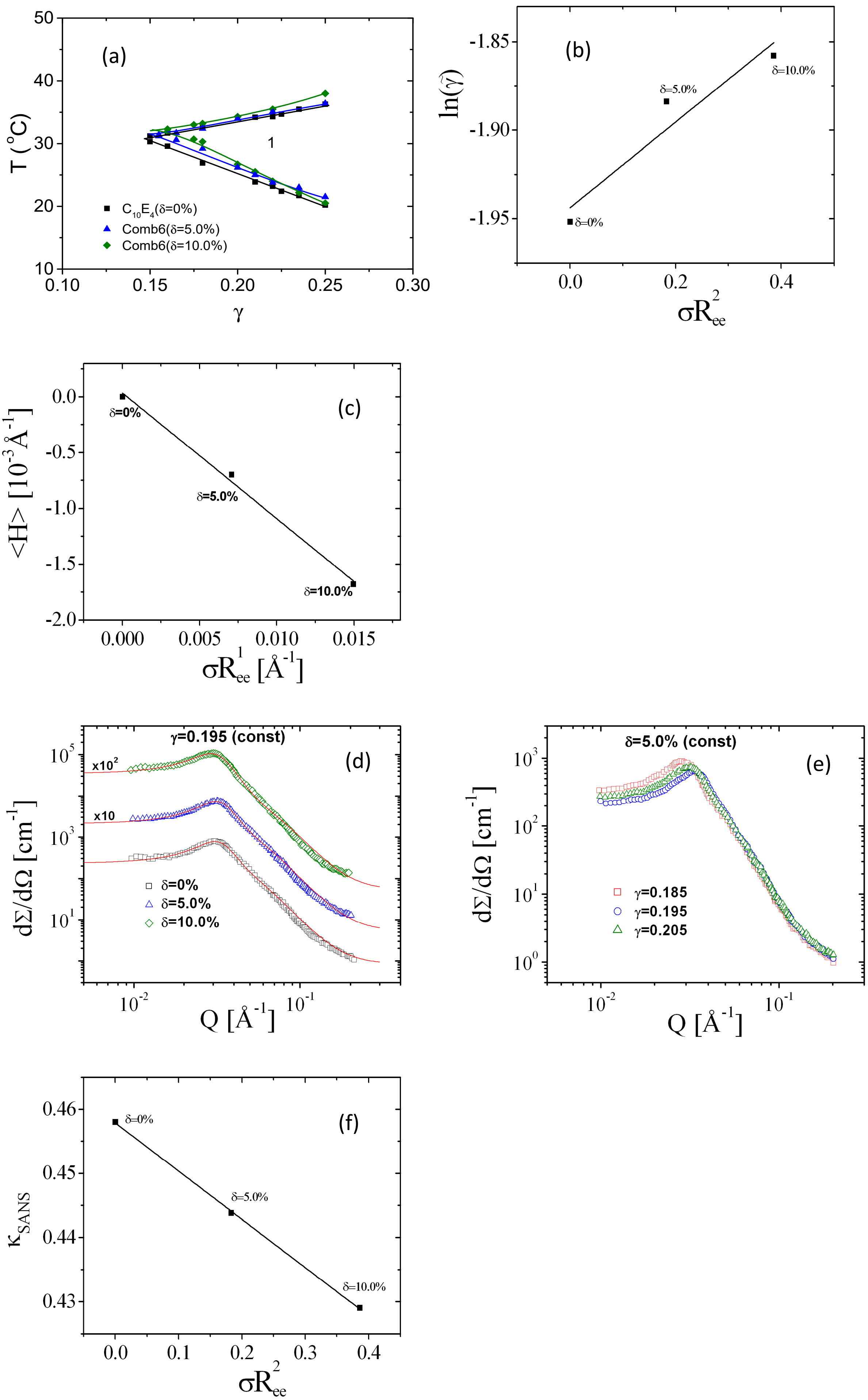

Fig. S7 a-f: Microemulsion characterization with Comb 6.

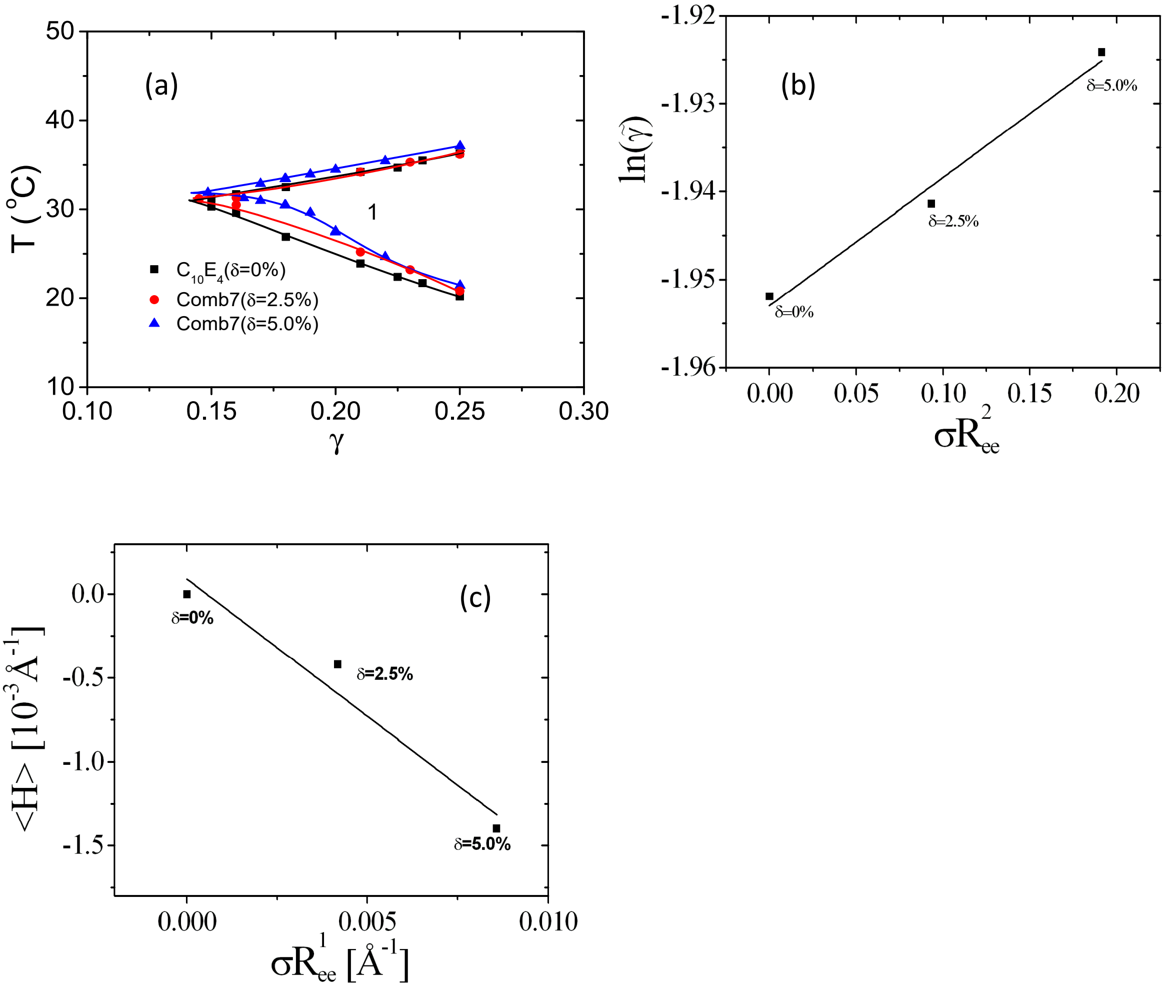

Fig. S8 a-f: Microemulsion characterization with Comb 7. SANS data were not measured for this sample.

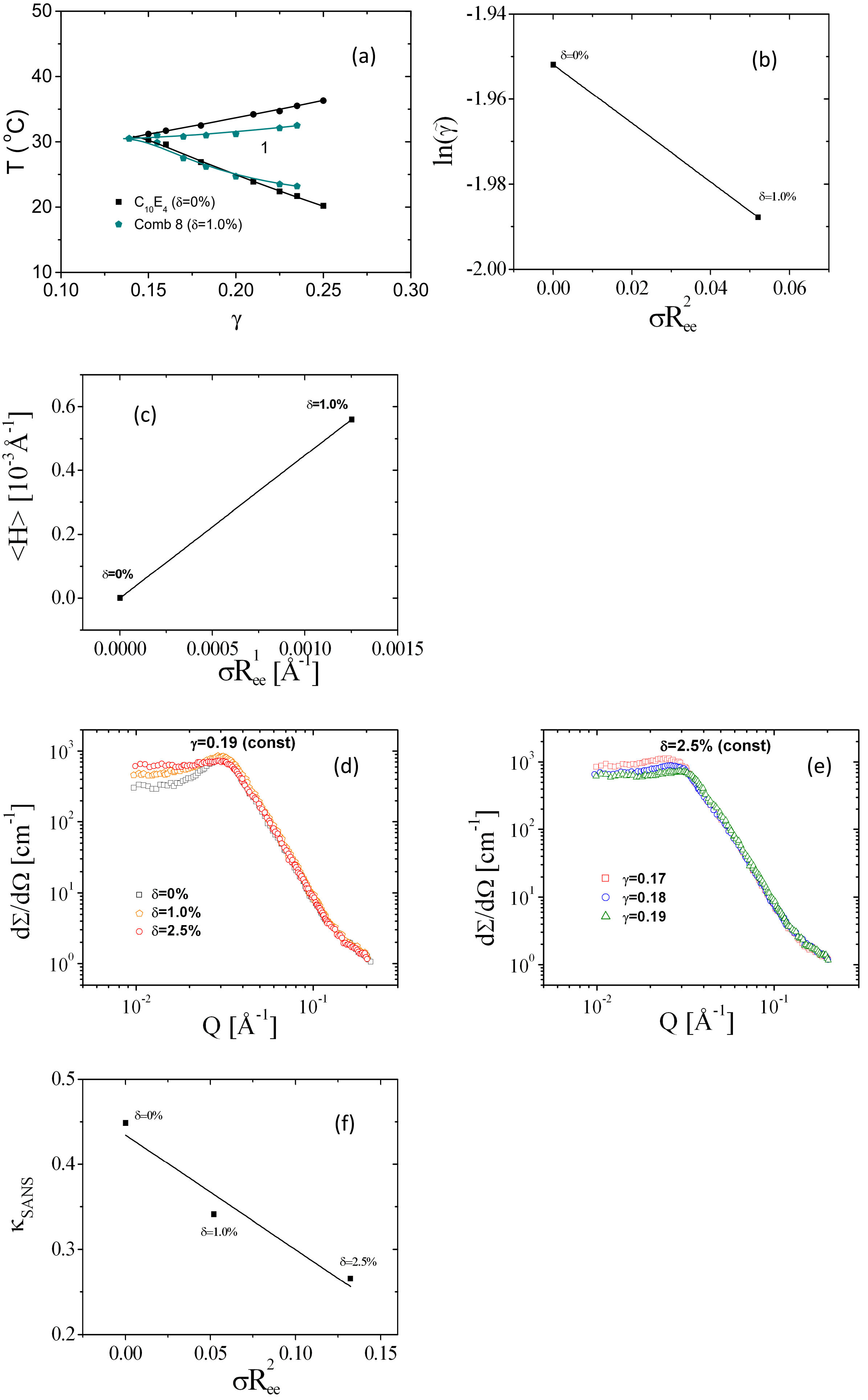

Fig. S9 a-f: Microemulsion characterization with Comb 8.

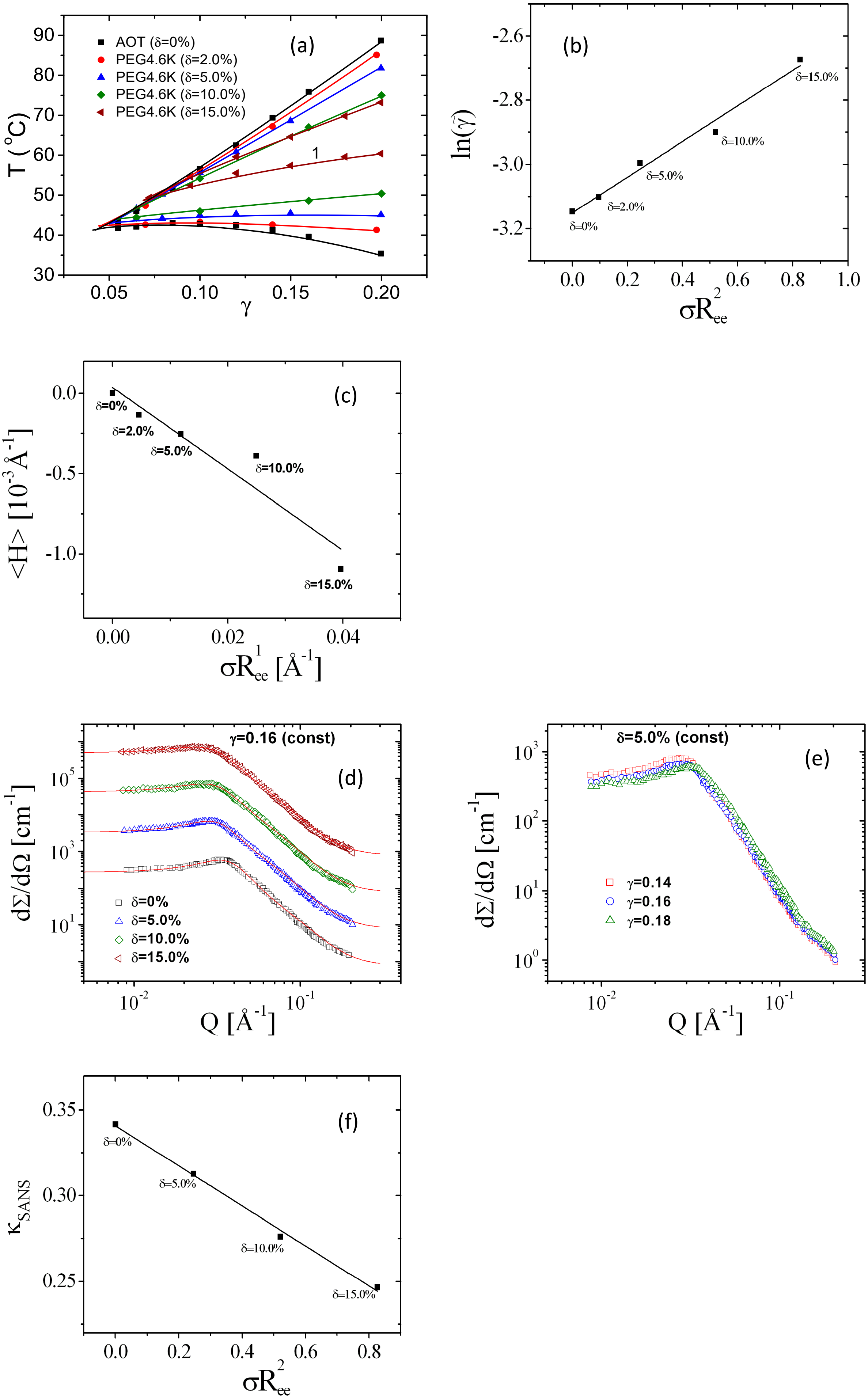

Fig. S10 a-f: Microemulsion characterization with PEG in AOT system.

To facilitate the formation of a one-phase microemulsion with the surfactant AOT, we added 0.6 wt% NaCl to the aqueous phase. This weakens the repulsive forces between the head groups and suppresses the tendency to the system form a lamellar phase. This salt concentration is kept constant throughout the study for the PEG/AOT system.

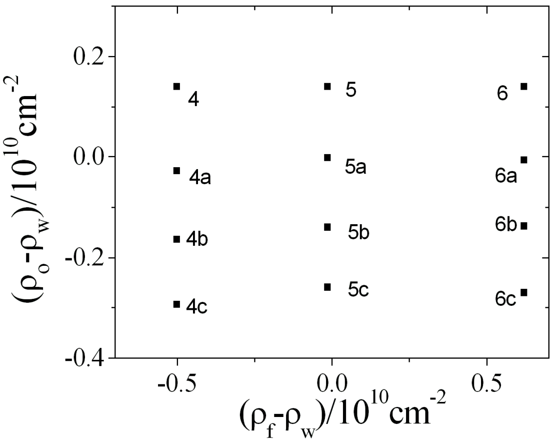

Fig. S11: The selected contrasts for the contrast variation experiments on Comb 4 in the microemulsion. Not shown here are the bulk and film contrast conditions. For the points descending the y-axis, protonated oil was sequentially added to the microemulsion. The samples 4-6 were separate samples.

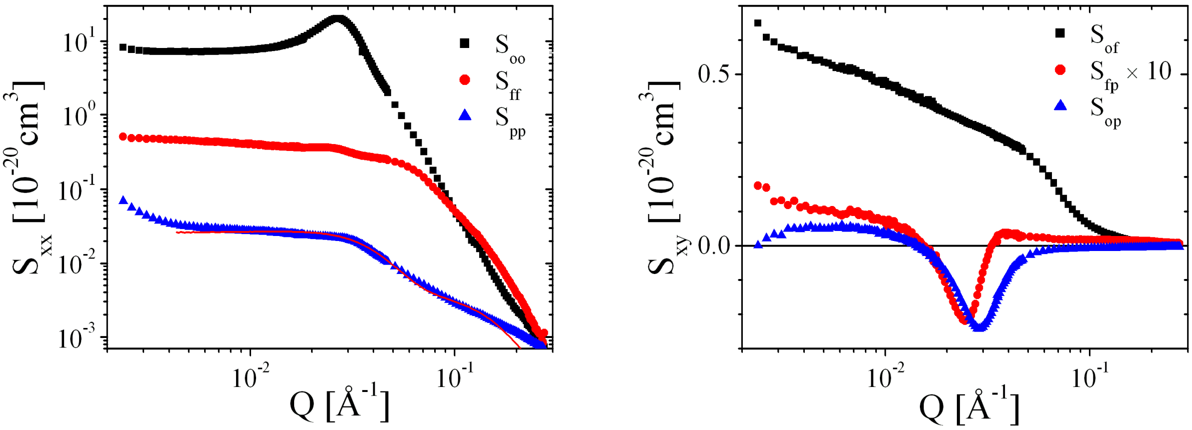

Fig. S12 a-b: Contrast variation – Singular value decomposition of partial scattering functions for the Comb 6 (compare Fig. 9a/b). A model fit for the polymer scattering is represented by the red line.

The decomposition of different correlation functions for the Comb 6 polymer (Fig. S10) looks rather similar to that for Comb 4 (Fig. 9a/b). The polymer-polymer correlation is approx. 2 times weaker than estimated from the molar mass of the polymer. This could be a result of the rather long, interfering sidechains, losing their correlation upon contact. The polymer-polymer correlation could be described by a non-stretched structure along the membrane as described by eqs. 16 to 25. The second hint for lost correlations is the reduced sidechain size by a factor of approx. 1.45. So the whole Comb 6 may be rather collapsed in the microemulsion (Fig. S11). The water-polymer and film-polymer cross-correlations display a clear correlation peak around Q = 0.02 $Å^{-1}$ to 0.03 $Å^{-1}$. This is thought to be related to the polymer-film compartments that inflate the water domains due to the repulsive interactions between the sidechains of different comb polymers. The negative sign of the correlation peaks indicates that the oil domains at the polymer position are compressed to rather small thicknesses, much smaller than the equilibrium thickness. This situation is quantitatively different from the weaker interaction of sidechains of Comb 4.

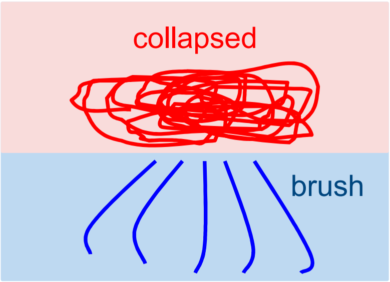

Fig. S13: Sketch of the possible collapsed conformation of the Comb 6 polymer in the microemulsion.

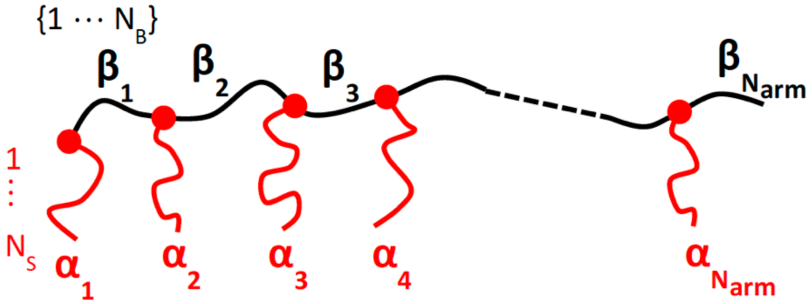

Fig. S14: Sketch of the polymer scattering calculus in a good or theta solvent. It is assumed that all of the backbone fragments are of equal length and that all of sidechain fragments are of equal length. Note that, in contrast to the nomenclature in the main manuscript, the number of monomers in the side chain and the backbone fragment are here denoted N_S and N_B respectively. N_{arm} is the number of sidechains (arms).

In the following, we calculate the polymer scattering in a good or theta solvent, which means that backbone and sidechain are equally dissolved and have no preferences to the single solvent. The sidechain / sidechain correlation is calculated to be:

$$\begin{aligned} S_{S-S}(Q) &= \left\langle \sum_{r=1}^{N_{arm}} \sum_{s=1}^{N_{arm}} \sum_{i\in\alpha_r} \sum_{j\in\alpha_s} \exp\left(i\vec{Q}(\vec{r}_{r,i}-\vec{r}_{s,j})\right)\right\rangle \\ &= \sum_{r=1}^{N_{arm}} \sum_{s=1}^{N_{arm}} \sum_{i\in\alpha_r} \sum_{j\in\alpha_s} \exp\left(-\frac{1}{6}Q^2\ell^2\cdot\Delta(r,i,s,j)\right) \text{with } \omega := \exp\left(-\frac{1}{6}Q^2\ell^2\right) \\ &= \sum_{r\neq s}\sum_{i,j}\omega^{|r-s|N_B+i+j} \quad + \sum_{r=s}\sum_{i,j}\omega^{|i-j|} \\ &= \left(P(N_{arm},\omega^{N_B}) - N_{arm}\right)\cdot Q^2(N_S,\omega) \quad + \quad N_{arm}\cdot P(N_S,\omega) \end{aligned} \tag{S1}$$

where $\Delta(r,i,s,j)$ describes the monomeric distance of two monomers and the two functions are defined as:

$$P(N,\omega) = \left((N-1)\frac{1+\omega}{1-\omega} - 2\omega\frac{1-\omega^{N-1}}{(1-\omega)^2}\right)\cdot\left(\frac{N}{N-1}\right)^2 \tag{S2}$$

$$Q(N,\omega) = \frac{1-\omega^N}{1-\omega} \tag{S3}$$

The indices r and s indicate the rth and s^{th} side chain of the polymer and ℓ is the length of a single bond. For the backbone, the classical polymer scattering was obtained. Here, the starting point was a simple form

factor of an ideal polymer chain and the other possible information for the comb polymers was included in the function step by step. This can be presented the following way:

$$S_{B-B}(Q) = P(N_{arm} \cdot N_B, \omega) \tag{S4}$$

For the cross-term, one can obtain:

$$S_{B-S}(Q) = \sum_{r,s} \sum_{i \in \beta_r, j \in \alpha_s} \omega^{\Delta_2(r,i,s,j)} \tag{S5}$$

$$\text{with} \quad \Delta_2(r,i,s,j) = \begin{cases} (s-r)N_B + i + j & \text{for } s \geq r \\ (r-s-1)N_B + i + j & \text{for } s < r \end{cases}$$

$$= \frac{(\omega^{N_B}+1)\omega^{N_{arm}N_B} - 2(N_{arm}-1)(\omega^{N_B}-1) - 3\omega^{N_B} + 1}{(\omega^{N_B}-1)^2}$$

$$\cdot Q(N_B, \omega) \cdot Q(N_S, \omega)$$

For the overall Polymer scattering in solution one can obtain,

$$\frac{d\Sigma}{d\Omega}(Q) = \frac{\phi_{polymer}}{V_{polymer}} \cdot \tag{S6}$$

$$\left((\Delta\rho_S v_S)^2 S_{S-S} + (\Delta\rho_B v_B)^2 S_{B-B} + 2(\Delta\rho_B v_B)(\Delta\rho_S v_S) S_{B-S}\right)$$

$$\cdot J_1^2(Q \cdot d) \cdot \frac{\sin(Q \cdot l/2)}{Q \cdot l/2}$$

The leading prefactor contains the polymer concentration and the total polymer volume of a single molecule. The monomeric volumes occur as v_S, and v_B. The corresponding contrasts in solution are $\Delta\rho_S$ and $\Delta\rho_B$. N_{arm} is the number of sidechains (arms), while N_S and N_B denote the number of monomers in one fragment. The last factors describe a monomer shaped as a cylinder (with diameter *d*, and length *l*). Here the low-Q Taylor expansions are sufficient to deal with the experimental Q-range.

Fig. S15: RI traces of the SEC measurements of Combs 1 to 7. The signal at a retention time of 29.3 min., labeled with ‘F’, represents the flow rate marker.

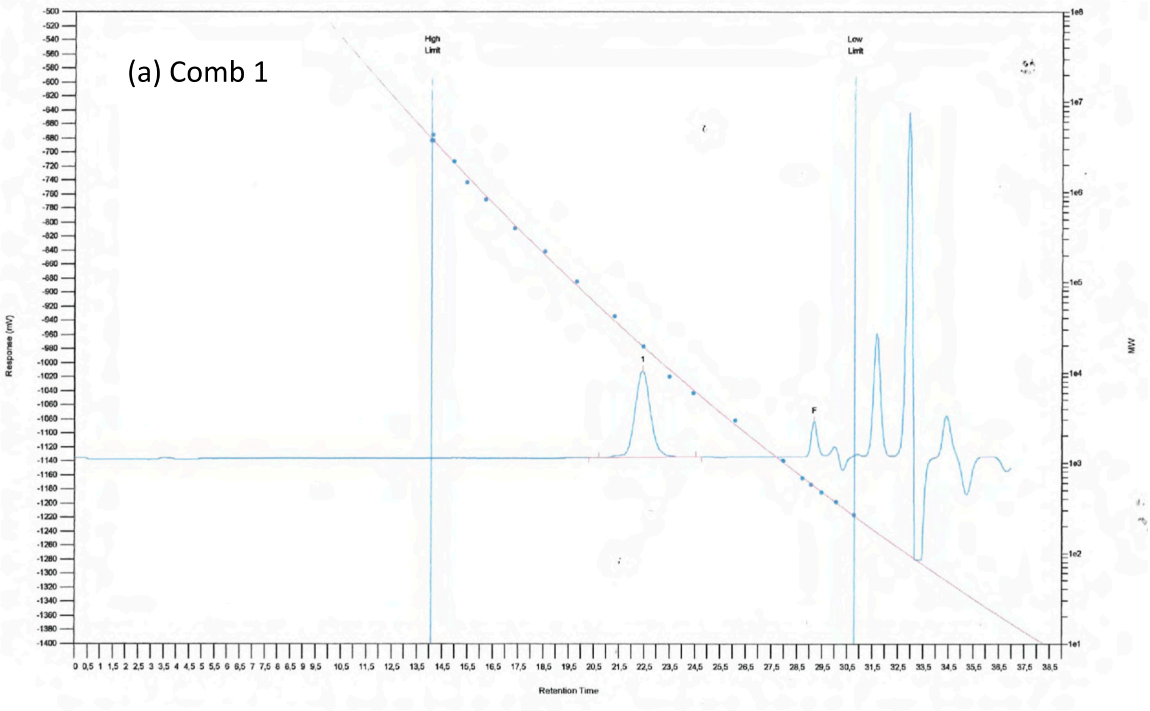

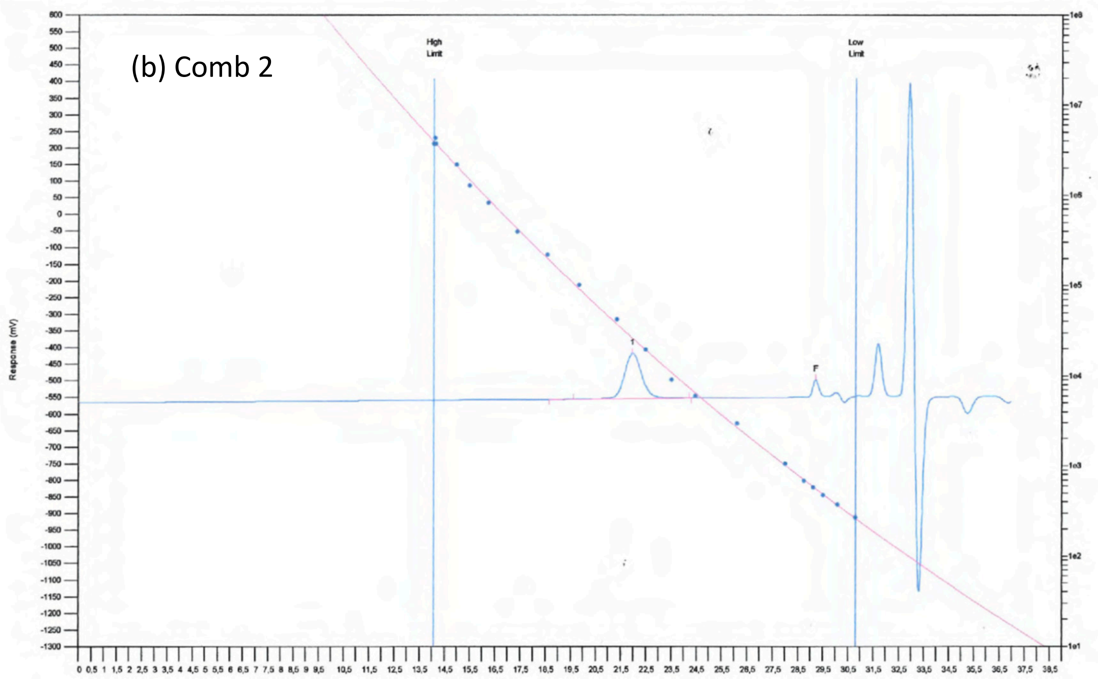

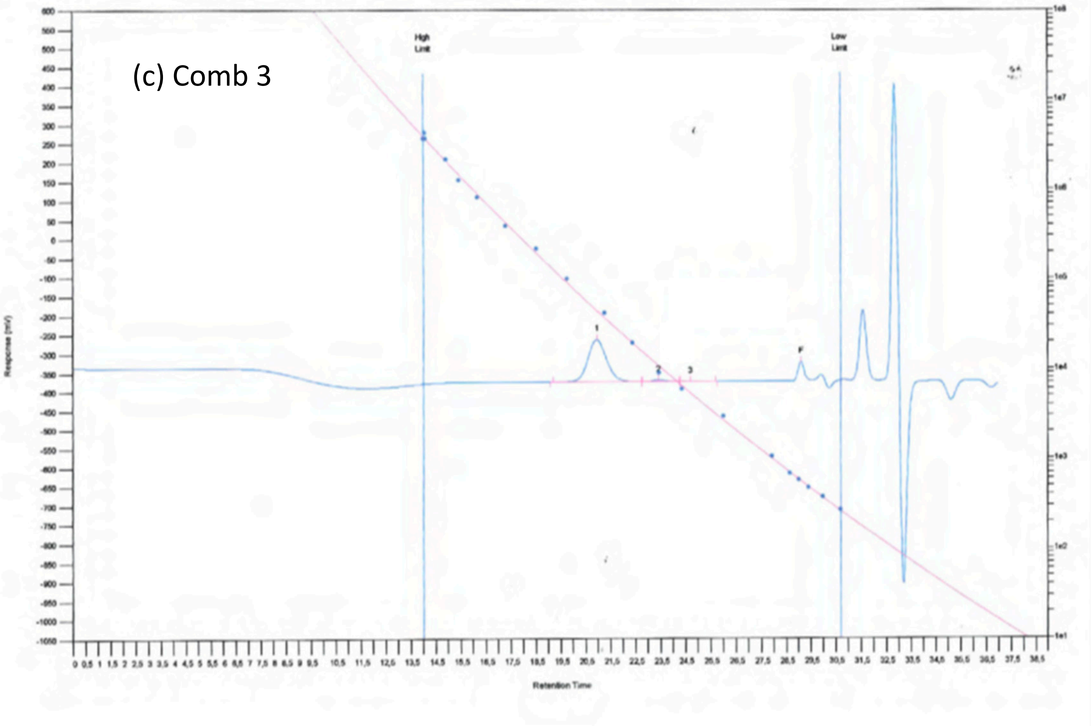
(c) Comb 3
High Limit
Low Limit
1
2
3
F
Response (mV)
Retention Time

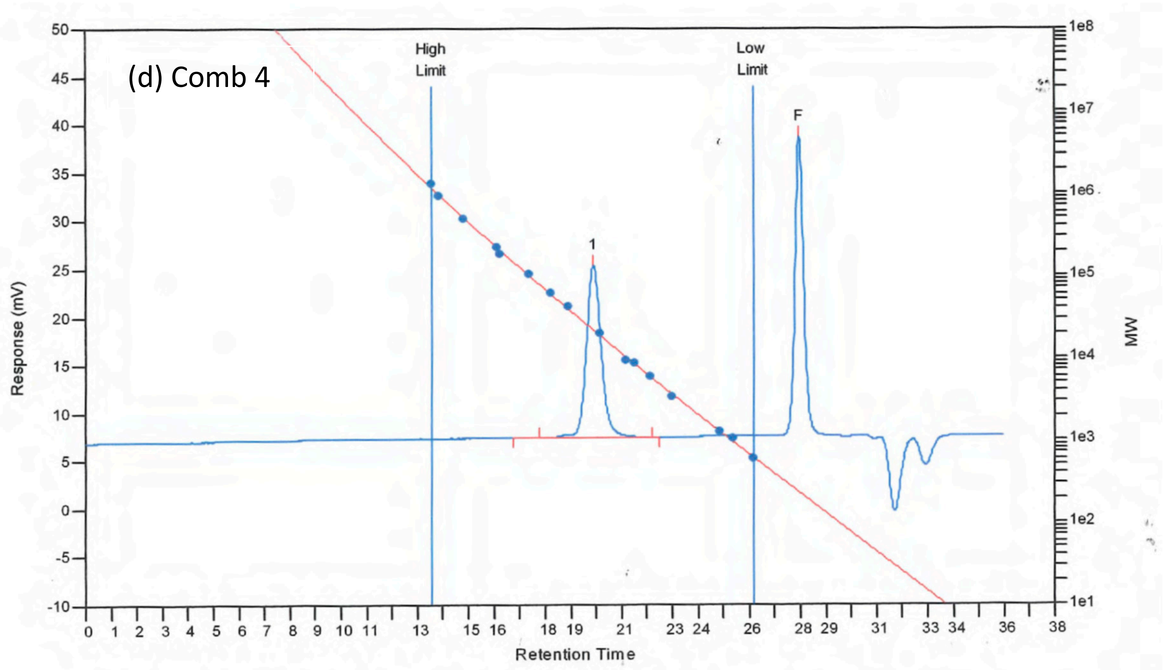
(d) Comb 4
High Limit
Low Limit
1
F
Response (mV)
MW
Retention Time
1e8
1e7
1e6
1e5
1e4
1e3
1e2
1e1

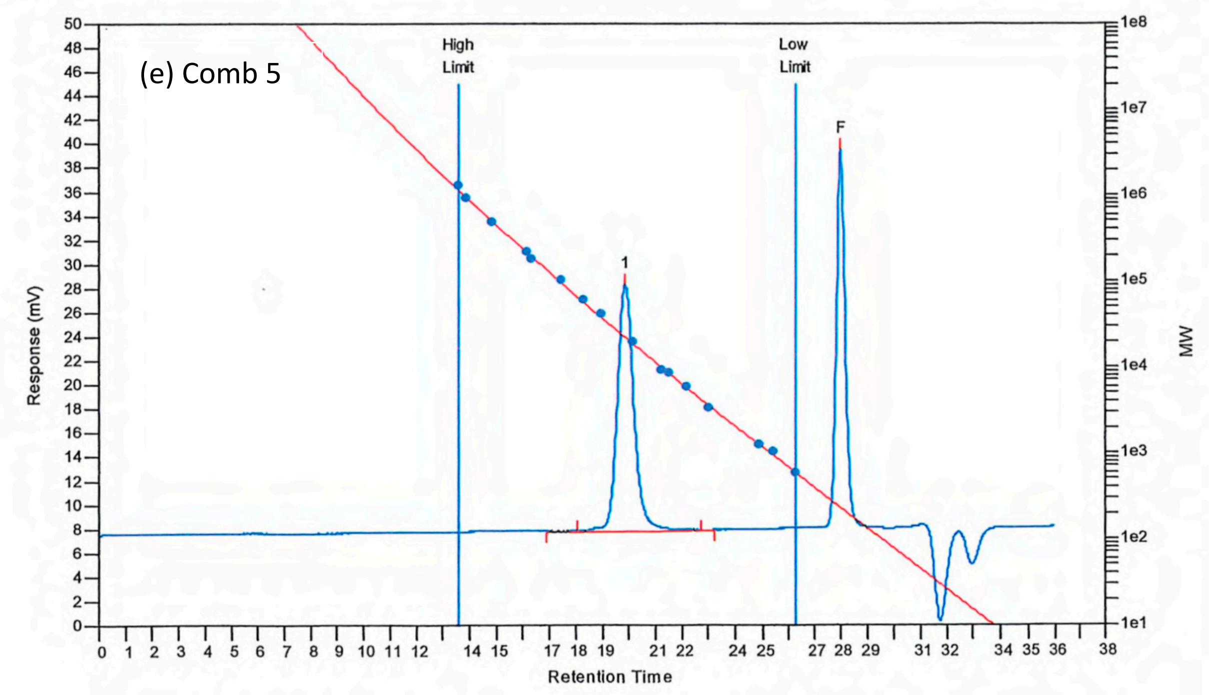
(e) Comb 5
High Limit
Low Limit
F
1
Response (mV)
Retention Time
MW
1e8
1e7
1e6
1e5
1e4
1e3
1e2
1e1

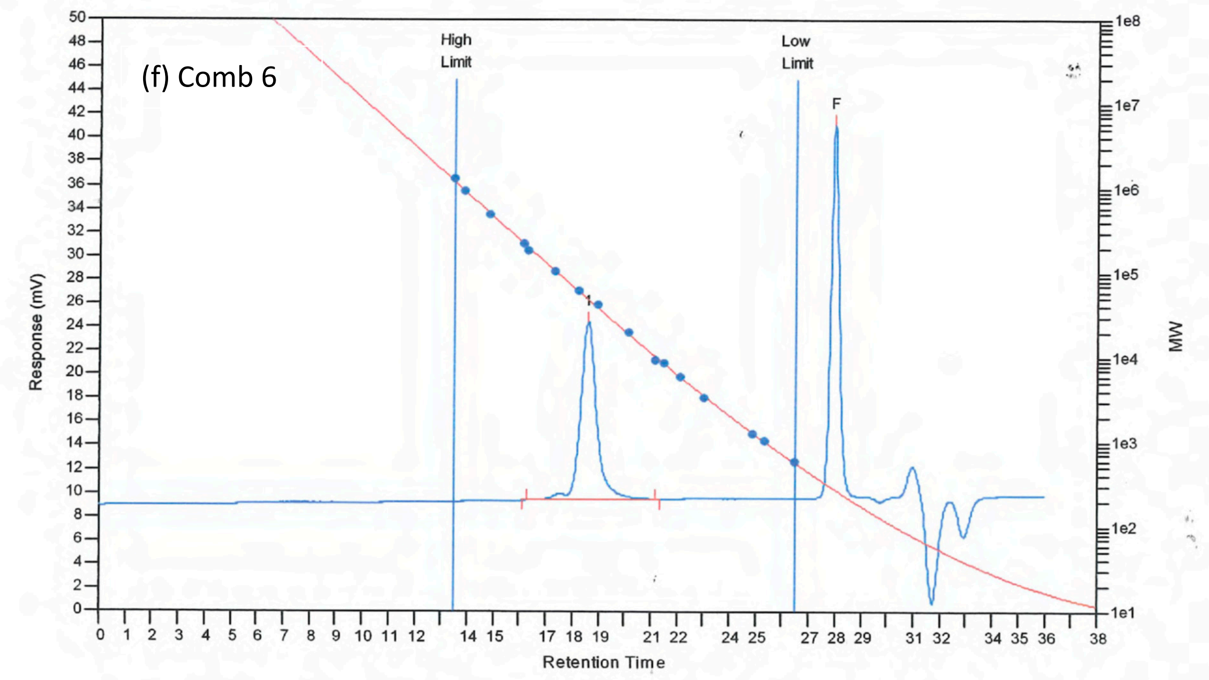
(f) Comb 6
High Limit
Low Limit
F
1
Response (mV)
Retention Time
MW
1e8
1e7
1e6
1e5
1e4
1e3
1e2
1e1

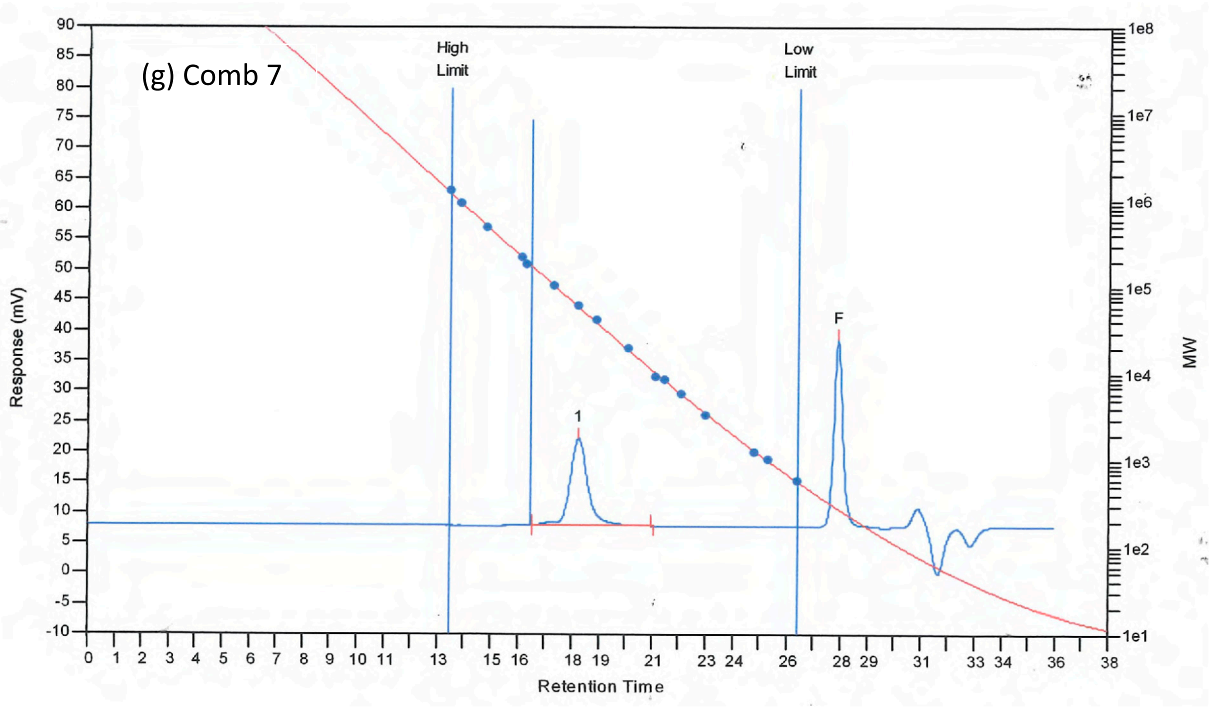
(g) Comb 7
High Limit
Low Limit
Response (mV)
MW
Retention Time
1
F

Fig. S16. ^{1}H-NMR spectra of the comb polymers 1-8 (a-h).

(a) Comb 1

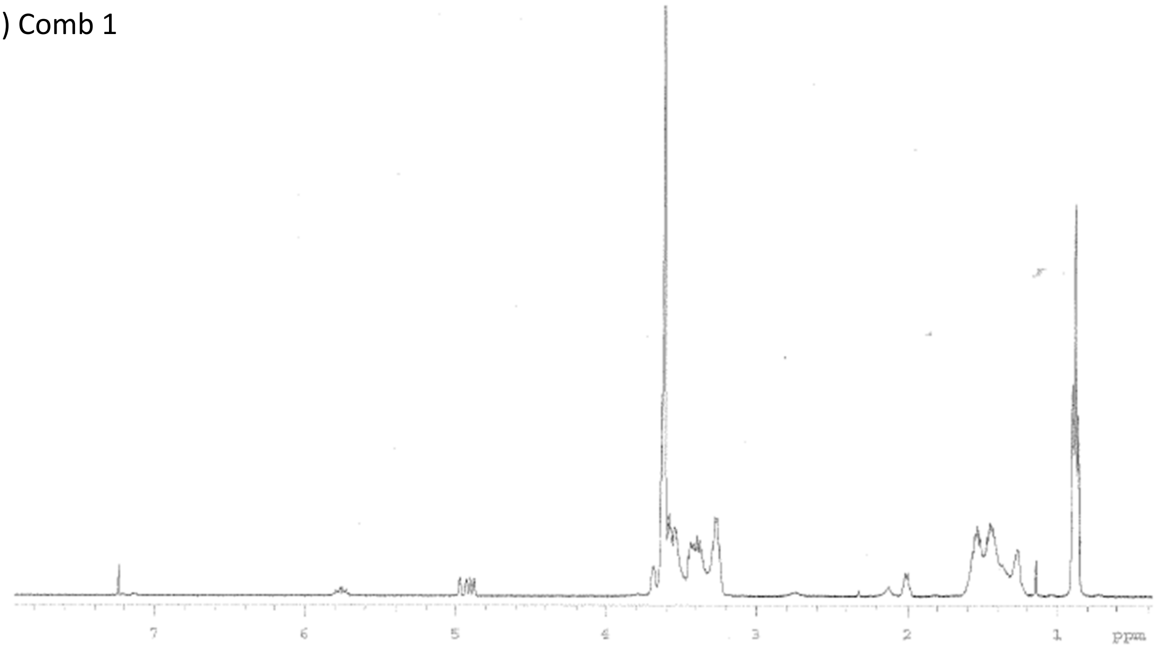

(b) Comb 2

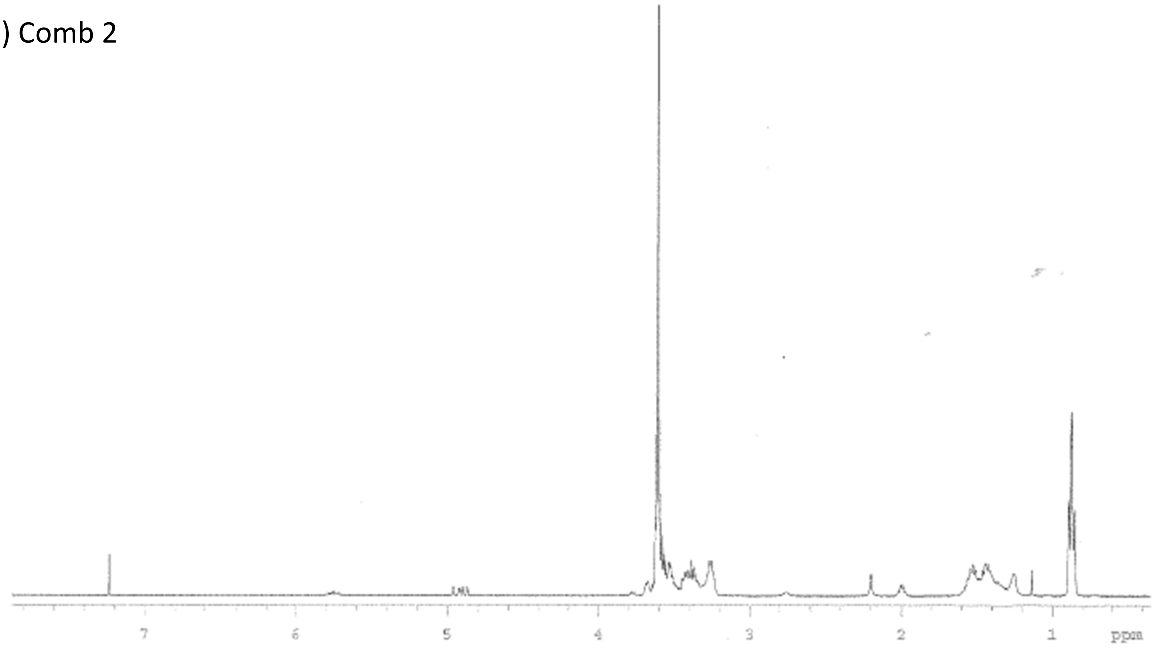

(c) Comb 3

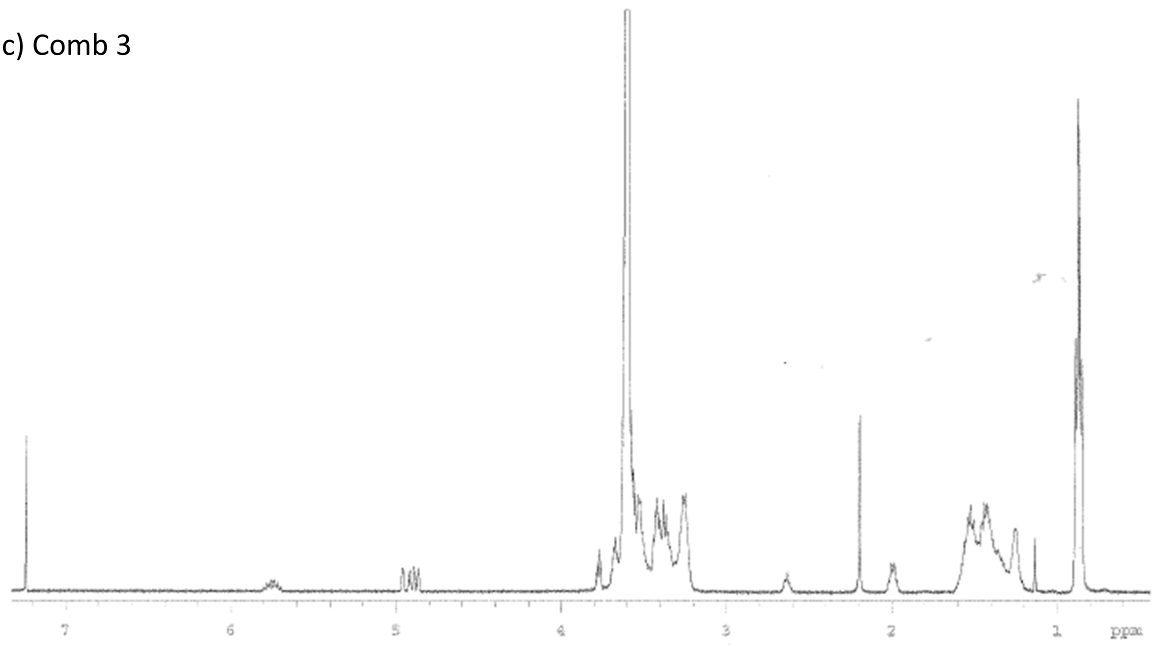

(d) Comb 4

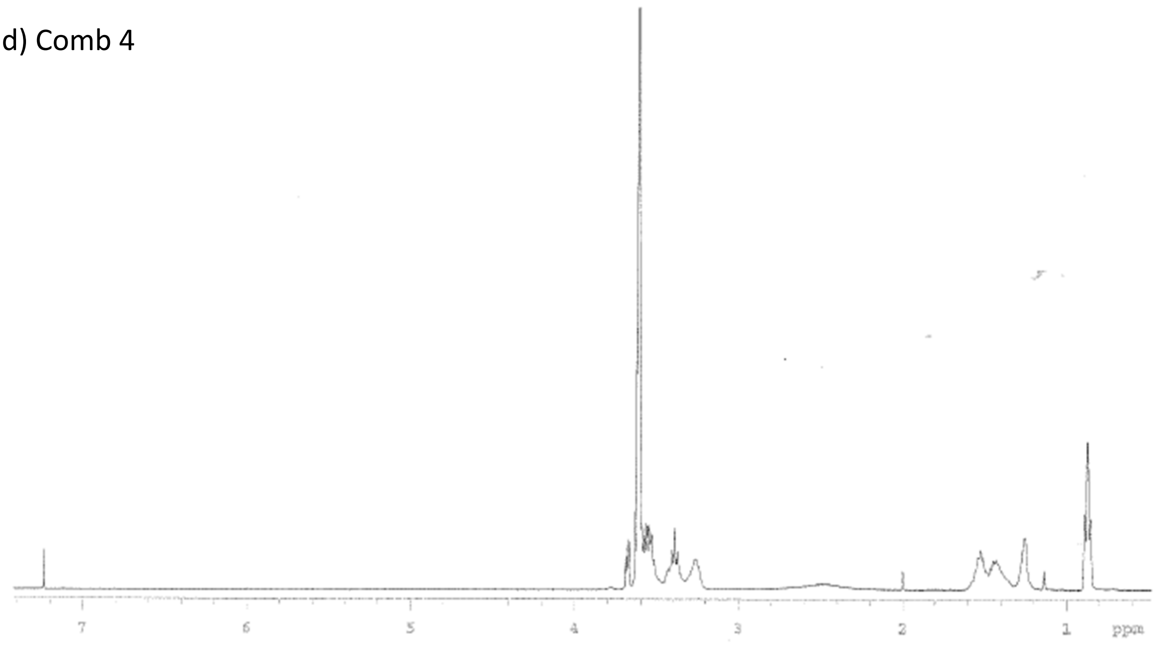

(e) Comb 5

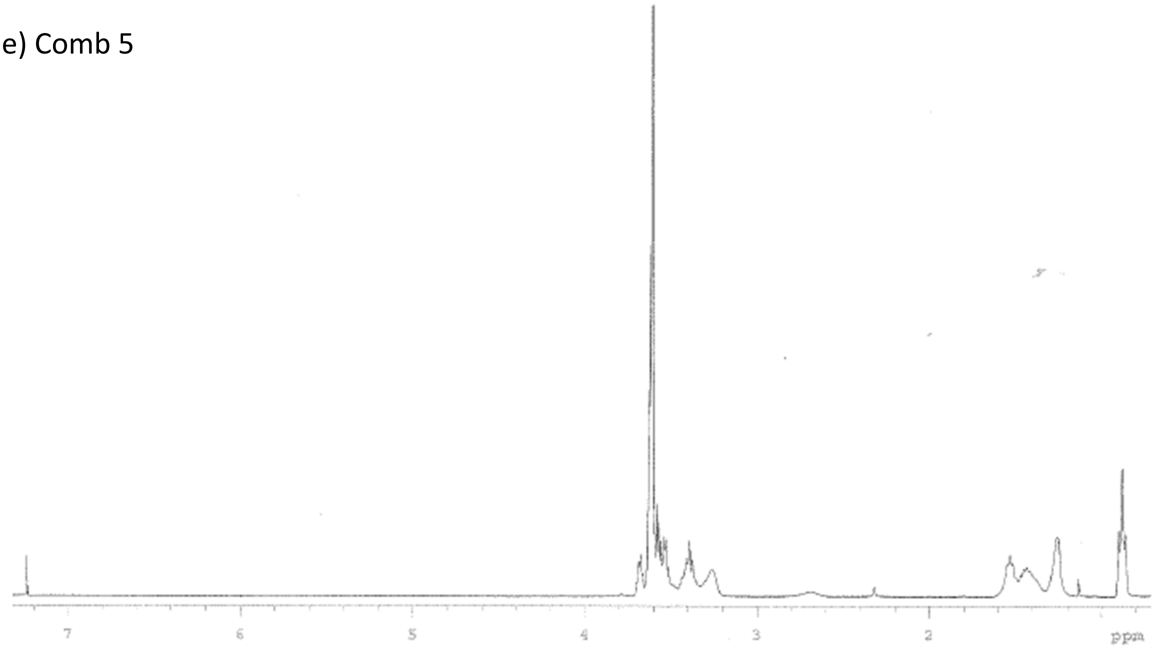

(f) Comb 6

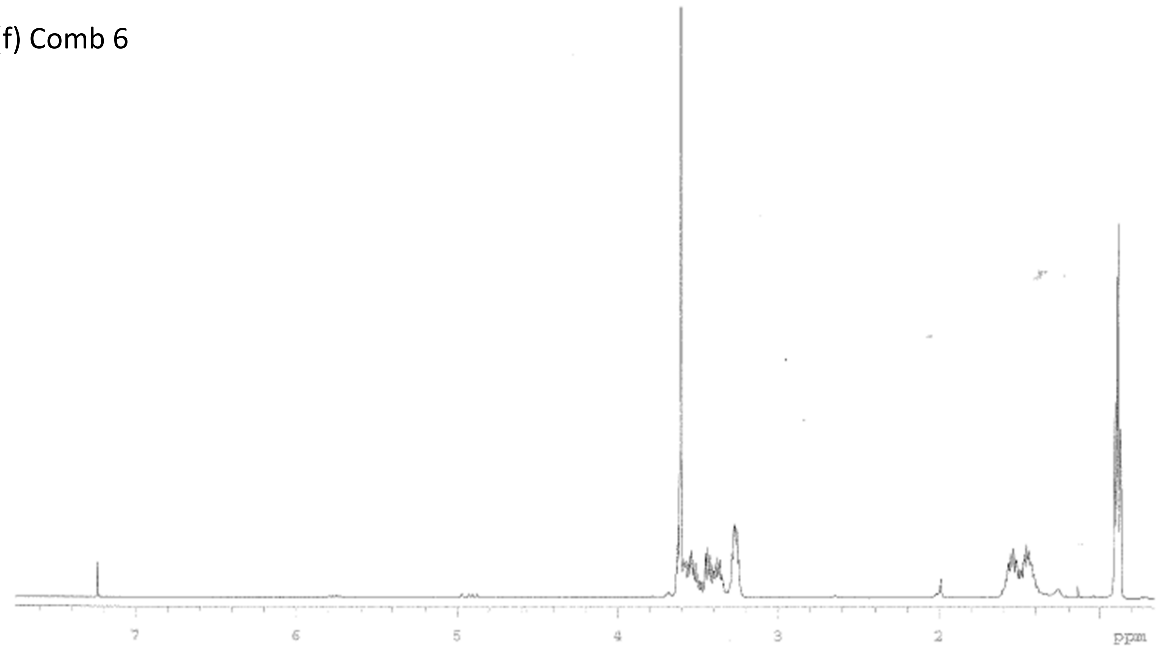

(g) Comb 7

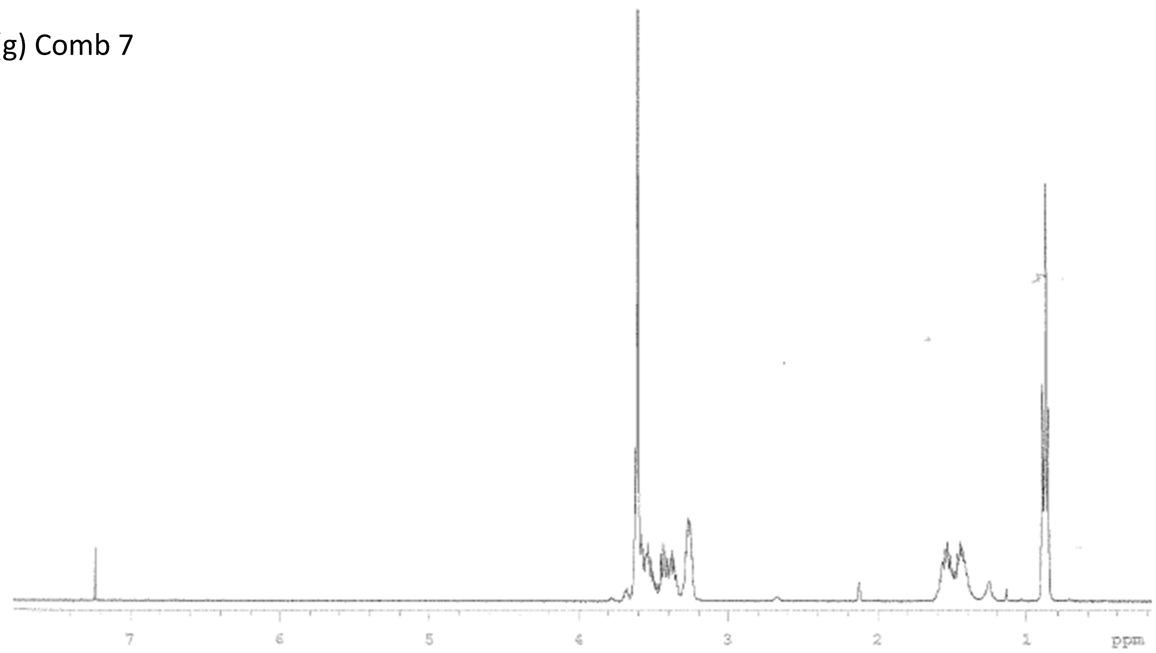

(h) Comb 8

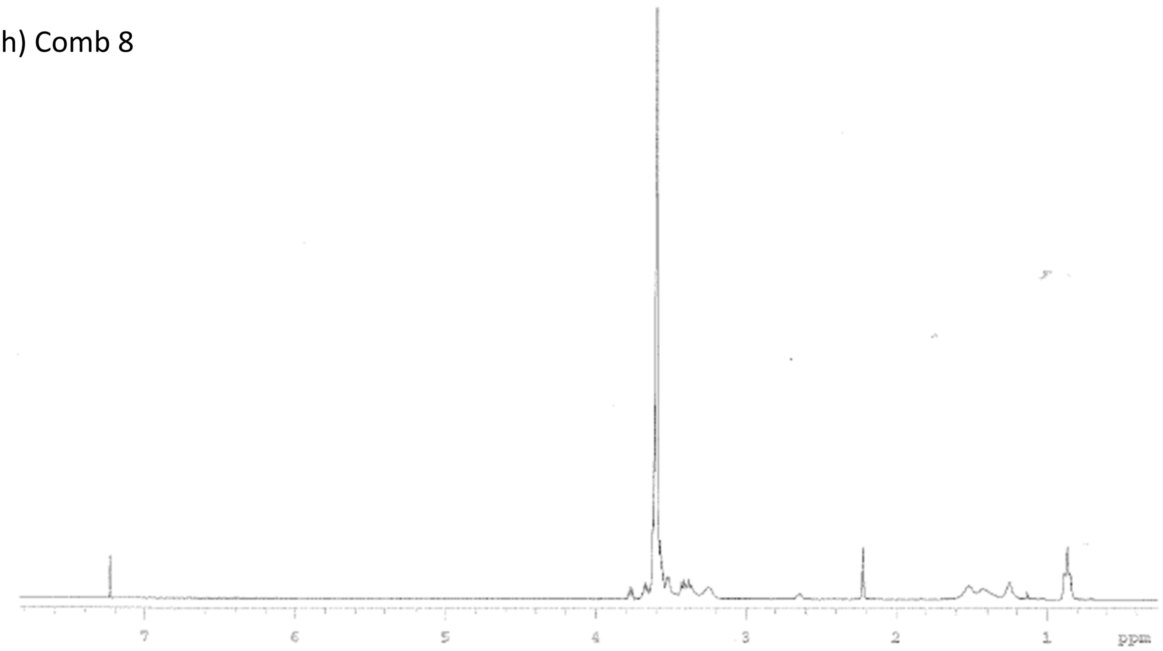

References

[1] Allgaier, J.; Hövelmann, C.H.; Wei, Z.; Staropoli, M.; Pyckhout-Hintzen, W.; Lühmann, N.; Willbold, S. Synthesis and rheological behavior of poly (1, 2-butylene oxide) based supramolecular architectures. *RSC advances* **2016**, *6*, 6093-6106. https://doi.org/10.1039/C5RA24547H

[2]Allgaier, J.;Willbold, S.; Chang, T. Synthesis of Hydrophobic Poly (alkylene oxide)s and Amphiphilic Poly (alkylene oxide) Block Copolymers. *Macromolecules* **2007**, *40*, 518-525. https://doi.org/10.1021/ma062417g
